# Visceral Leishmaniasis in Pediatrics: A Case Series and a Narrative Review with Global Insights

**DOI:** 10.3390/tropicalmed10050136

**Published:** 2025-05-17

**Authors:** Valentina Andreottola, Chiara Santucci, Tommaso Bellini, Simona Matarese, Francesca Canzoneri, Gianluca Dell’Orso, Martina Finetti, Francesca Fioredda, Alessio Mesini, Emanuela Piccotti

**Affiliations:** 1 Department of Neuroscience, Rehabilitation, Ophthalmology, Genetics, Maternal and Child Health (DINOGMI), University of Genoa, 16146 Genoa, Italy; 5292002@studenti.unige.it (V.A.); 4487429@studenti.unige.it (C.S.); 2 Pediatric Emergency Room and Emergency Medicine Unit, Emergency Department, IRCCS Istituto Giannina Gaslini, 16147 Genoa, Italy; simonamatarese@gaslini.org (S.M.); francescacanzoneri@gaslini.org (F.C.); martinafinetti@gaslini.org (M.F.); emanuelapiccotti@gaslini.org (E.P.); 3 Hematology Unit, Hemato-Oncological Department, IRCCS Istituto Giannina Gaslini, 16147 Genoa, Italy; gianlucadellorso@gaslini.org (G.D.); francescafioredda@gaslini.org (F.F.); 4 Infectious Diseases Unit and COVID Hospital, Department of Pediatrics, IRCCS Istituto Giannina Gaslini, 16147 Genoa, Italy; alessiomesini@gaslini.org

**Keywords:** bone marrow aspiration, hemophagocytic lymphohistiocytosis, liposomal amphotericin B, neglected tropical diseases, pediatric emergency department, vector control, visceral leishmaniasis, zoonosis

## Abstract

Visceral leishmaniasis (VL) is a severe parasitic disease caused by *Leishmania* spp., with a significant impact on pediatric populations, particularly in endemic regions. The diagnosis of VL in children requires a high index of suspicion, as clinical manifestations—such as prolonged fever, hepatosplenomegaly, and pancytopenia—overlap with other infectious and hematologic diseases. While serological and molecular tests aid in detection, bone marrow aspiration remains the gold standard for definitive diagnosis. In this case series, we describe five pediatric patients diagnosed with VL in Italy, emphasizing the importance of a timely and accurate diagnostic approach. Liposomal amphotericin B (LAmB) is the first-line treatment in Southern Europe due to its high efficacy and reduced toxicity. Our patients received a standard regimen of 3 mg/kg daily for five days, plus an additional dose on day 10, leading to rapid clinical improvement. However, some cases required supportive care, such as red blood cell transfusions, particularly in patients with co-infections. Despite being a neglected disease, VL is re-emerging in Europe, influenced by climate change, increased pet ownership, and migration from endemic regions. Prevention strategies focus on vector control, canine vaccination, and public health awareness. The global rise in pediatric VL highlights the need for improved surveillance, access to affordable treatments, and the development of effective vaccines to mitigate the disease burden in both endemic and non-endemic areas.

## 1. Introduction

Leishmaniasis is one of the most dangerous and neglected infectious diseases and is caused by protozoans of the *Leishmania* complex [1,2,3,4,5,6,7]. This parasitic infection may affect various animal species, particularly dogs, which are the main reservoirs of the disease [6,8,9]. Other domestic and wild mammals may also be reservoirs, including cats, rodents, and hares [7]. The disease is primarily transmitted through the bites of female blood-feeding insects known as sandflies or phlebotomines, which inhabit the Mediterranean, tropical, and subtropical regions [1,4,5,8,10,11,12,13]. Through sandfly bites, which act as vectors, the infection can potentially be transmitted from dogs to humans, causing leishmaniasis to be classified as a zoonosis [6,7]. There are over 50 described species of Leishmania, and nearly 20 are pathogenic to humans [9,14]. The global incidence of leishmaniasis varies by region, historical period, and disease form, with an increasing trend in Europe [1,2,7,8,13,15,16]. Each year, the World Health Organization (WHO) reports 700,000–1 million new cases worldwide, with visceral leishmaniasis (VL) accounting for approximately 50,000–90,000 of these cases [2,17,18]. The disease remains endemic to more than 90 countries, with the highest burden observed in South Asia, East Africa, and South America; however, sporadic cases and outbreaks are increasingly reported in regions where leishmaniasis is rare, including southern and central Europe [6,8,10].

Leishmaniasis has long been classified as a neglected tropical disease, but its epidemiological landscape is evolving owing to multiple factors. Among these, climate change plays a significant role in expanding the geographical range of *Leishmania* vectors, allowing the establishment of endemic foci in previously non-endemic areas such as Central and Northern Europe [5,6,7,10,15,19,20,21,22]. Additionally, human migration, urbanization, and deforestation are other factors that may have contributed to increased human exposure to infected sandflies. The growing population of immunocompromised individuals, including those with human immunodeficiency virus (HIV) infection, malignancies, or those undergoing immunosuppressive therapy, has also led to an increase in severe and atypical VL cases [8,9,10]. In the last 25 years, there has been a global increase in VL incidence, with a shift in transmission dynamics observed in endemic and non-endemic areas alike [1,6,18].

The disease may manifest as cutaneous, mucocutaneous, or visceral leishmaniasis [3,15]. VL, the most severe form, is fatal if left untreated because of progressive immune dysfunction, hematologic abnormalities, and multiorgan involvement. In pediatric cases, the clinical presentation of VL can vary and is not easy to diagnose [2,6,7,17]. However, it commonly includes persistent or recurrent high fever lasting several weeks, and it may be associated with weight loss, lymphadenopathy, and hepatosplenomegaly [2,6,7,17]. Other clinical features may include pancytopenia, severe anemia, and hypergammaglobulinemia, mimicking hematologic malignancies [7,13,19].

In this study, we aimed to evaluate the clinical and epidemiological characteristics of VL in a tertiary hospital during the previous season, providing a narrative review of the etiology, pathophysiology, diagnosis, and treatment of this re-emerging disease.

## 2. Materials and Methods

This is a retrospective, observational study conducted at IRCCS Istituto Giannina Gaslini, a third-level Children’s Hospital located in Genoa, Italy. We reported clinical cases of VL ≤ 14 years old of VL encountered between 1 January and 31 December 2024, at the Pediatric Emergency Department (PED). Clinical, epidemiological, and microbiological data were retrospectively collected and analyzed. A systematic literature search was conducted using PubMed to identify relevant articles. In addition to PubMed, reference lists of relevant articles were manually screened to expand the search coverage. Three researchers independently conducted the search to ensure comprehensive coverage and included keywords related to leishmaniasis, vector-borne transmission, epidemiology, pathogenesis, clinical manifestations, diagnostic methods, treatment, and prevention strategies. Articles published over the last 10 years were included in this search. Search terms were selected based on their relevance to the research question and were combined using Boolean operators. Filters were used to limit the search to articles written in English and published in peer-reviewed journals. Additionally, manual searches were performed by screening the reference lists of the relevant articles. Duplicates were identified and removed using a reference management software, and any discrepancies in article selection were resolved through consensus. The final selection of articles was based on their relevance to the research question and the quality of evidence presented. Data extraction was performed independently by three reviewers and crosschecked to ensure accuracy.

## 3. Results

We identified five clinical cases, three males and two females, with a median age of 24 months (range 15–72). The incidence was 1.35 per 10,000 PED visits, and *L. infantum* was detected in 100% of patients. Of the five cases, one patient was from the province of Genoa (GE), one was from the province of Savona (SV), and the remaining three were from the province of Imperia (IM).

No patient reported visiting other VL-endemic areas in the 12 months preceding the onset of symptoms. None of the patients had immunodeficiency due to other chronic disorders. The median number of days needed to establish a conclusive diagnosis since the onset of symptoms was 17 (8–30). Below, there is a brief description of each individual clinical case. Table 1 presents the main blood test results. Bold values indicate measurements outside the normal reference range.

### 3.1. Case 1

A previously healthy two-year-old boy was transferred to our PED from a regional hospital due to persistent fever for approximately one month and trilinear cytopenia, recently accompanied by cough, diarrhea, and vomiting. The patient resided in Sanremo (IM), a rural area, and the family owned outdoor animals. Upon arrival at the PED, blood tests revealed anemia, thrombocytopenia, leukopenia, and hypergammaglobulinemia. Abdominal ultrasonography revealed splenomegaly. The patient was admitted for further diagnostic investigations, including serological tests for Epstein–Barr virus (EBV), cytomegalovirus (CMV), Toxoplasma, and Parvovirus B19, and a peripheral blood smear. All tests were negative. A bone marrow aspirate was then obtained, and optical microscopy showed no evidence of *Leishmania* or signs suggestive of oncological diseases. However, a real-time Polymerase Chain Reaction (RT-PCR) test for protozoan DNA in bone marrow blood conducted at the National Institute of Health (ISS) in Rome returned positive results, leading to a definitive diagnosis. The patient was treated with liposomal amphotericin B (LAmB) at a dose of 3 mg/kg daily for five days, followed by a single additional dose on day 10. Upon initiation of therapy, rapid improvement in the patient’s general clinical condition was observed on day three of treatment, with prompt normalization of body temperature and gradual improvement of laboratory parameters. During hospitalization, the patient required a transfusion of red blood cells and intravenous administration of albumin and potassium.

### 3.2. Case 2

A previously healthy three-year-old female resident of Diano Marina (IM) was admitted to our PED because of persistent fever, anorexia, and fatigue lasting for approximately 20 days, associated with upper respiratory tract symptoms. The baseline blood tests showed pancytopenia. The family reported no known contact with dogs, and they were residents of an urban area. Abdominal ultrasound showed splenomegaly, and viral serologies for EBV, CMV, Parvovirus B19, and adenovirus were negative. Bone marrow aspirate showed no evidence of hemato-oncological disease or protozoa. This may be due to the low parasitic burden or sampling error, as microscopy sensitivity is limited compared to RT-PCR. However, bone marrow blood samples were sent to the ISS in Rome, confirming the diagnosis of VL. The patient was treated with LAmB at a dose of 3 mg/kg daily for five days, followed by a single additional dose on day 10. Upon initiation of therapy, a rapid improvement in the patient’s general clinical condition was observed on day two of treatment, with prompt normalization of body temperature and gradual improvement of laboratory parameters. During hospitalization, the patient required two red blood cell transfusions and intravenous administration of albumin and potassium.

### 3.3. Case 3

A previously healthy 15-month-old boy presented to a spoke hospital with persistent fever lasting for 13 days, poor response to acetaminophen, accompanied by cough and faint, self-resolving pinpoint rash. He was a resident of Albissola Marina (SV) in an urban area and reported no known contact with dogs. Baseline blood tests showed pancytopenia and slightly elevated inflammatory marker values. The blood smear was normal, and the Toxoplasma serology test results were negative. Further serological tests were not performed. Due to a failed response to antibiotics, a bone marrow aspirate was obtained, and optical microscopy showed hematopoietic cells at all maturation stages, hemophagocytic histiocytes, and evidence of protozoa, compatible with a diagnosis of VL, later confirmed by the RT-PCR results from ISS. Abdominal ultrasonography revealed hepatosplenomegaly. The patient was treated with LAmB at a dose of 3 mg/kg daily for 5 days, followed by a single additional dose on day 10. Upon initiation of therapy, a rapid improvement in the patient’s general clinical condition was observed on day four of treatment, with prompt normalization of body temperature and gradual improvement of laboratory parameters.

### 3.4. Case 4

A two-year-old female resident of Rapallo (GE) was admitted to our PED due to eight days of persistent fever, accompanied by a non-pruritic erythematous facial rash, cough, rhinitis, and exudative pharyngitis. Antigenic rapid EBV tests were negative. The family lived in a rural area and owned two dogs affected by canine leishmaniasis. Blood tests revealed anemia, piastrinopenia, and other signs of early hemophagocytic lymphohistiocytosis (HLH). Serological tests for EBV, CMV, and Parvovirus B19 were negative. Abdominal ultrasonography revealed hepatosplenomegaly. Suspecting VL, a bone marrow aspirate was obtained and sent to the ISS in Rome for RT-PCR to detect protozoan DNA. Both the microscopic observations and RT-PCR results were consistent with the proposed diagnosis. The patient was treated with LAmB at a dose of 3 mg/kg daily for 5 days, followed by a single additional dose on day 10. Upon initiation of therapy, a rapid improvement in the patient’s general clinical condition was observed on day three of treatment, with prompt normalization of body temperature and gradual improvement of laboratory parameters.

### 3.5. Case 5

A previously healthy six-year-old boy, resident of Castel Vittorio (IM), was transferred to our PED from a spoke hospital due to pancytopenia, persistent fever for approximately 15 days, diarrhea, and vomiting. The family reported no known contact with the dogs. Moreover, they resided in an urban area. Serological tests for EBV, CMV, and Toxoplasma were negative, but Parvovirus B19 was positive. Abdominal ultrasonography revealed splenomegaly, and a peripheral blood smear showed no abnormalities. Due to persistent fever and worsening clinical conditions, a bone marrow aspirate was performed, which revealed protozoa and allowed the diagnosis of VL, which was later confirmed by the RT-PCR results from the ISS. The patient was treated with LAmB (Ambisome) at a dose of 3 mg/kg daily for 5 days, followed by a single additional dose on day 10. Upon initiation of therapy, a rapid improvement in the patient’s general clinical condition was observed on day two of treatment, with prompt normalization of body temperature and gradual improvement of laboratory parameters. During hospitalization, the patient required a red blood cell transfusion with intravenous administration of albumin and potassium.

## 4. Discussion

This study reports and describes the clinical and epidemiological characteristics of VL observed and diagnosed in our PED, focusing on clinical features. The five identified cases represent an outbreak, considering that in the post-COVID era, we did not have any cases of VL at our PED. For greater clarity, we structured the discussion of the literature review in the following subsections.

### 4.1. Etiology and Incidence

Historically considered a problem in tropical and subtropical regions, leishmaniasis has increasingly become a concern in Western nations, including Italy [10,19,20]. Although various *Leishmania* species exist, the only species considered native to Europe, and thus responsible for nearly all cases of European Leishmaniasis, is *Leishmania infantum*, transmitted through canine reservoirs [1,3,8,9,16,18,20,22]. However, isolated cases of *Leishmania tropica* and *Leishmania donovani* have been reported due to immigration between African, Middle Eastern, and European regions [5,13,14,15,20,23,24]. Moreover, there has recently been a notable expansion of the disease into areas previously considered unaffected, involving inland and mountain areas, and regions in northern Europe, leading the WHO to consider leishmaniasis as an endemic disease in most European countries [1,12,14,17,18,19]. After the COVID era, an increased incidence of VL has been observed. This linear increase in incidence cannot be fully explained by active case surveillance and improvements in RT-PCR diagnostics technique [1,12,15].

These numbers will continue to rise owing to increasing human mobility, both for travel and migration, in previously non-endemic regions [2,4,5,8,12,14,15,19,20]. The incidence of symptomatic infection increases in individuals with specific risk factors, and in Europe, 90% of infections manifest in the pediatric population [3,13]. Other high-risk populations are immunocompromised patients due to HIV/*Leishmania* coinfection or patients who underwent chemotherapy or treatment with biological drugs [15,19,20,21,25].

All the reported cases involved *L. infantum* infection. All cases were autochthonous, occurring in Italian children or in children permanently residing in Italy, specifically in the Liguria region, with no history of travel to other endemic areas. Only two of the five patients lived in a rural area and had domestic or farm animals. Despite residing in urban areas, sandfly vectors may be present, and infection is acquired via their bites.

### 4.2. Pathophysiology

**Figure 1:**
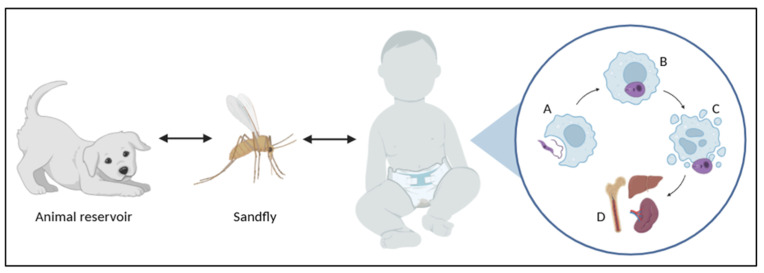
Schematic of *Leishmania infantum*. Life cycle: sandflies transmit promastigotes to humans and animal reservoirs. In the host, promastigotes are phagocytosed by macrophages (A) and differentiate into amastigotes (B). Amastigote replication leads to macrophage lysis (C) and dissemination mainly to reticuloendothelial organs—spleen, liver, and bone marrow (D)—causing VL manifestations.

Transmission occurs through the bite of infected sandflies, which inoculate the promastigote form, the flagellated and motile stage of the parasite present in the vector, into the vertebrate host [1,2,3]. Promastigotes are rapidly phagocytosed by macrophages and other phagocytic immune cells within which they transform into amastigotes [3,8]. Amastigotes represent an intracellular form of the parasite and actively proliferate within macrophages, causing cell destruction and enabling disease progression to other host cells and tissues [1,2,3,8]. The ability of *Leishmania* to survive and replicate within macrophages is a key factor in its pathogenesis and is facilitated by evasion mechanisms that inhibit the host immune response, such as suppression of reactive oxygen species production and interference with antigen-presentation pathways [7]. When a sandfly feeds on the blood of an infected host, it ingests macrophages containing amastigotes. In the vector gut, amastigotes undergo further morphological transformations, culminating in promastigotes that migrate to the proboscis of the sandfly, ready to be transmitted to a new host [3]. Parasite development within the sandfly is influenced by environmental factors such as temperature and humidity, which can affect vector competence and transmission dynamics [8]. Once inside the host, *Leishmania* modulates the immune response to establish chronic infection. The parasite induces a shift toward a T-helper 2 (Th2) immune response, leading to the production of anti-inflammatory cytokines, such as interleukin-10 (IL-10) and transforming growth factor-beta, which suppress macrophage activation and hinder parasite clearance [5,8]. Additionally, the parasite can exploit host lipid metabolism by residing within the lipid-rich compartments of macrophages, further enhancing its survival [7,8]. The incubation period of the disease can vary from a few days to several months or even years. Factors influencing clinical onset include parasite strain, host immune status, and coexisting infections, which may modulate disease severity and progression [3,7,8]. In immunocompromised individuals, such as those with HIV coinfection, *Leishmania* can cause more aggressive disease manifestations with higher parasitic loads and an increased risk of relapse despite treatment [5,7]. Figure 1 shows the life cycle of the parasites.

### 4.3. Clinical Manifestations and Laboratory Findings

Human Leishmaniasis may present in different clinical forms, and its diagnosis may be challenging [6,15,16]. Symptoms caused by this protozoan infection may vary according to the interaction between the host immune response and infectious features of *Leishmania* spp. [7,22]. Cutaneous Leishmaniasis (CL) is the most common form and manifests as numerous ulcerated lesions on exposed parts of the body, often leaving permanent scars [3,15]. Mucocutaneous Leishmaniasis is a severe form of CL characterized by destructive lesions in the mucous membranes of the nose, mouth, and oral cavity. Finally, VL, also known as kala-azar, is the most severe form of VL, and if untreated, it has a nearly 100% mortality rate [3,17,22]. Moreover, VL is the most common form in children and in countries of the Western Mediterranean basin, including Italy [4,5,10,19,20,22]. VL may be 100% fatal within two years of infection due to the direct effects of the protozoan on the spleen, liver, and bone marrow, and the high risk of secondary infections and hemorrhages associated with cytopenia [15]. Asymptomatic carriers of the infection can remain asymptomatic for years, but may develop symptomatic disease if they experience immunosuppression from any cause [19,20,21,25]. Subsequently, the disease may present as acute, subacute, or indolent forms.

In pediatric cases, the clinical presentation of VL can vary greatly; however, it commonly includes persistent or recurrent high fever lasting several weeks, associated with weight loss, lymphadenopathy, and hepatosplenomegaly [2,6,7,17,22]. Other symptoms may be specific to fatigue and irritability and, in some cases, may include gastrointestinal manifestations, such as diarrhea and vomiting [22]. The main blood test finding is cytopenia, most frequently involving red blood cells and platelets, and less commonly, white blood cells [7,17,22]. Thus, affected patients may exhibit typical symptoms related to deficits in each blood cell line, including fatigue, pallor, cyanosis, tachycardia, headache, ecchymosis, petechiae, spontaneous or prolonged bleeding, and recurrent, severe, and/or opportunistic infections [16,17,22]. Other laboratory test findings may reveal hypoalbuminemia, hypergammaglobulinemia, hypertransaminasemia, and elevated markers of inflammation, such as C-reactive protein (CRP) and erythrocyte sedimentation rate [7,16,17].

These findings are a direct consequence of the pathogenesis of the disease, which primarily affects macrophages, where *Leishmania* parasites reside and multiply as amastigotes [6,9]. In children, an immature immune system exacerbates susceptibility to disease progression, which may explain why VL is more common in pediatric age [6,7,21]. Thus, the pathogenesis is marked by severe immune dysregulation, resulting in hypergammaglobulinemia and impaired cellular immunity, which increases the risk of secondary infections [21]. Clinical and laboratory findings are nonspecific and overlap with many other infectious and hematologic–oncologic diseases, which may be more common than VL [6,11,16,17,22].

In our case series, all patients presented with persistent fever, with a mean duration of 17 days prior to medical evaluation, ranging from 8 to 30 days. Fatigue and irritability were consistently observed in all the cases. Gastrointestinal symptoms were reported in two patients, while upper respiratory manifestations were present in four cases. Skin rash was documented in two patients. Laboratory findings revealed that all patients exhibited anemia and thrombocytopenia, and leukopenia was observed in four patients, and CRP levels were elevated in all cases. Hypergammaglobulinemia and hypoalbuminemia were detected in all patients. Abdominal ultrasound findings showed hepatomegaly in two patients and splenomegaly in all patients. These data suggest that in patients with persistent fever, the clinical suspicion of leishmaniasis should always be considered, and that blood tests may represent a valuable tool to confirm the diagnosis.

### 4.4. Diagnosis

The diagnosis of VL in pediatric patients requires a high index of clinical suspicion supported by laboratory tests and imaging [2,18]. A thorough medical history and epidemiological knowledge are crucial to guide diagnostic suspicion and determine which tests should be prioritized. A comprehensive physical examination, blood tests, abdominal ultrasound to detect visceromegaly, and serological tests for the most common viral infections are essential [15]. In endemic areas, pediatricians often rely on a combination of clinical evaluation (fever, splenomegaly, anemia) and laboratory findings (pancytopenia, elevated globulins) [7,16,17]. Although invasive, microscopic identification of Leishman–Donovan bodies in bone marrow aspirates remains the gold standard [22]. RT-PCR is particularly useful for detecting low parasite burden, a common scenario in asymptomatic or early-stage cases [12,15,22]. In endemic countries, especially rural areas, asymptomatic carriers are common but difficult to estimate, and seropositivity does not necessarily indicate symptomatic disease [22]. In contrast, blood samples have low sensitivity, except for HIV patients who may present with higher parasitemia [22].

There are direct methods, including visualization and culture of the protozoan on a blood smear test, and indirect methods that detect Leishmania-specific antibodies in the blood or parasite DNA through RT-PCR on blood samples [15]. These investigations can be performed on peripheral blood, bone marrow aspirate, bone marrow biopsy, spleen biopsy, liver biopsy, lymph node biopsy, or, in cases of CL, skin biopsy or scraping. The detection of anti-leishmanial antibodies or leishmanial antigens is a less invasive test, but their sensitivity and specificity may be low because antibodies decrease slowly after the infection and are also present in asymptomatic infected patients [15,22]. RT-PCR has the highest sensitivity and specificity, whereas serological testing and microscopic observation of blood often yield false negatives [12,15,22]. Thus, given the suboptimal sensitivity and specificity of some methods, it is highly recommended to combine different diagnostic tests with the same or different tissue samples [15,22]. The gold standard for diagnosing leishmaniasis is the identification of amastigotes by microscopic analysis of tissue samples. In cases of VL, bone marrow aspiration may be indicated, as analyses performed on bone marrow samples provide the best risk–benefit ratio with good sensitivity and specificity. In all our cases, RT-PCR was performed on DNA extracted from bone marrow aspirate samples, and molecular confirmation by RT-PCR performed at the ISS in Rome supported the diagnosis.

In the clinical cases we presented, the suspicion of VL was initially based on the clinical presentation characterized by persistent fever, cytopenia, and visceromegaly, accompanied by hypergammaglobulinemia, features common to all patients. In three out of five patients, there was direct microscopic observation of the parasite, whereas RT-PCR testing resulted in all five patients being positive. From the time of admission to our institution, a diagnosis of VL was established within a mean of three days. These findings further support that, despite its invasive nature, bone marrow aspiration remains an essential diagnostic tool in pediatric VL, particularly when initial non-invasive investigations fail to yield definitive results.

### 4.5. Treatment

The treatment of pediatric VL varies depending on the *Leishmania* species involved, regional drug resistance patterns, and drug availability [15,16,22,26]. In Southern Europe, including Italy, LAmB is the preferred first-line therapy due to its high efficacy and reduced nephrotoxicity compared to conventional amphotericin B [2,7,15,17,22,26]. The standard regimen consists of daily intravenous administration at 3 mg/kg for five days, followed by a single additional dose on day 10, ensuring rapid parasite clearance while minimizing toxicity and hospital stay [7,15,22,26]. Alternative regimens, including single-day high-dose (10 mg/kg) or multi-day (3–5 mg/kg/day for 3–6 days) protocols, have been explored, achieving cumulative doses of 18–21 mg/kg [7,22,26,27]. However, the effectiveness of these protocols varies based on *Leishmania* species, with *L. infantum* often requiring higher cumulative doses than *L. donovani* [7]. In children, careful dose adjustments are essential to minimize adverse effects such as nephrotoxicity and electrolyte imbalances [7,26,27]. Although LAmB is preferred in high-income countries, in parts of Southeast Europe and other resource-limited regions, pentavalent antimonials such as meglumine antimoniate (Glucantim) are still used due to lower cost, despite their known toxicity and lower efficacy. Thus, although the availability of liposomal formulations in high-income countries has significantly improved patient outcomes, their high cost remains a major barrier in resource-limited settings [26]. To address these challenges, combination therapies—such as LAmB with miltefosine or paromomycin—are being explored to shorten treatment duration, reduce toxicity, and enhance efficacy. However, concerns regarding teratogenic risks and adherence issues continue to limit their widespread adoption [7,22]. Recently, adjuvant therapies to the currently used ones have been proposed, and among these, lupeol, a triterpenoid present in the flora of many edible plants, appears to be able to promote a balanced immune response, enhancing the body’s ability to combat *L. donovani* while potentially mitigating excessive inflammation [28]. The treatment regimen used in our cases followed the standard approach, with all patients receiving LAmB at 3 mg/kg daily for 5 days, plus a single additional dose on day 10. This protocol proved effective across a range of clinical presentations, from cases with mild pancytopenia to those with more severe hematologic involvement, including early laboratoristic signs of HLH (Case 4) and confirmed hemophagocytic histiocytes in the bone marrow (Case 3). The rapid clinical improvement observed in all cases, with normalization of fever and progressive recovery of hematologic parameters, further supports the efficacy of this regimen. However, three patients (Cases 1, 4, and 5) required red blood cell transfusions due to severe anemia, highlighting the need for supportive care in VL management. Additionally, the presence of Parvovirus B19 coinfection in Case 5 suggests that viral-induced bone marrow suppression can exacerbate VL-associated cytopenias, further complicating the clinical course and necessitating careful patient monitoring.

### 4.6. Prevention

In Italy and other European nations, prevention efforts are centered on zoonotic control, vector management, and public health awareness [7,10,19]. In Italy, Phlebotomus perniciosus is the primary vector, and *Leishmania infantum* is the predominant causative species [7,15,19,22]. Regular screening and treatment of domestic dogs with leishmanicidal drugs or vaccines can reduce transmission risk, and canine vaccination campaigns have been implemented in parts of Italy with moderate success [10]. Insecticide-treated nets, indoor residual spraying, and sandfly repellents are essential in endemic zones, and greater efforts must be made in vaccinating stray dogs, as they remain the primary source of transmission in many areas of the Mediterranean basin [5,10,11,12,13]. Finally, educating healthcare providers and the public about VL’s clinical signs ensures timely diagnosis and treatment [20].

A significant concern in Western countries is the re-emergence of VL among refugees and migrants from endemic regions [20,24]. These populations often face delayed diagnosis due to limited access to healthcare or cultural and language barriers [24]. According to our case series, other Italian and European hospitals have reported increasing cases among migrants from East Africa and the Middle East, highlighting the need for inclusive healthcare policies and targeted surveillance [3,19,24]. It therefore appears mandatory that eradication efforts are not only carried out in European countries that are facing the resurgence of Leishmaniasis, but also, and above all, in the regions that are historically most affected by the disease, namely, Eastern Africa [17,24]. Unfortunately, no control measures against sandflies are standardized, and, moreover, there is no vaccine available against human leishmania [7].

In Europe, the incidence of VL is influenced by factors like climate change, increased pet ownership, and migration from endemic regions [1,10,12,14,15,19,20]. Notably, the Mediterranean basin, including Italy, has seen a rise in pediatric VL cases, particularly in rural areas with high sandfly activity [1,5,7,10]. Despite being classified as a neglected disease, the increasing occurrence in Western nations demands greater public health attention. This shift underscores the necessity of a global perspective on this neglected tropical disease, particularly its impact on vulnerable populations such as children [2,4,6,8].

European people’s living conditions and urban infrastructure create fewer opportunities to be exposed to sandflies. Additionally, because Leishmaniasis in Europe is primarily caused by a single *Leishmania* species that mainly affects dogs, veterinary programs and awareness campaigns to control leishmaniasis in dogs are crucial to reduce the transmission risk to humans [10]. Moreover, the majority of the European population lives in favourable sanitary and socio-economic conditions, which are associated with better general health and a reduction in risk factors such as malnutrition, co-morbidities, co-infections, and more.

### 4.7. Differential Diagnosis

Due to the clinical similarities between VL, VL complications, and other conditions, the differential diagnosis must consider a broad spectrum of infectious and non-infectious diseases. Given that VL predominantly affects pediatric populations, especially in endemic regions, it is crucial for clinicians to include it in the differential diagnosis of children presenting with prolonged fever, hepatosplenomegaly, cytopenia, and signs of systemic inflammation [7].

Among infectious diseases, VL can mimic malarial infections, particularly in endemic regions where *Plasmodium* spp. can cause overlapping symptoms, such as fever, anemia, and splenomegaly. Typhoid fever and brucellosis also share similar systemic presentations, including persistent fever and hepatosplenomegaly, complicating their differentiation from VL. Additionally, tuberculosis may present with prolonged fever, weight loss, lymphadenopathy, and hepatosplenomegaly, making it a key differential diagnosis, particularly in immunocompromised individuals [29]. Considering infections with a higher incidence rate, it is important to rule out common viral infections in children that may present with a similar clinical picture, including CMV, EBV, and Parvovirus B19 infections.

VL shares numerous hematologic features with hematologic malignancies, including leukemia and lymphoma, as both can present with fever, pancytopenia, hepatosplenomegaly, and systemic inflammatory signs. This similarity can result in misdiagnosis, leading to unnecessary invasive diagnostic procedures or delays in the appropriate treatment. Bone marrow examination, along with molecular and serological testing, is essential for distinguishing between VL and malignancies.

Autoimmune and autoinflammatory conditions, such as systemic juvenile idiopathic arthritis-associated macrophage activation syndrome, can also mimic VL due to persistent fever, hepatosplenomegaly, pancytopenia, and high ferritin levels. In particular, MAS shares key pathophysiological mechanisms with HLH, a severe complication of VL, necessitating careful differentiation through molecular and histopathological studies [29].

Renal and hepatic involvement in VL may further complicate the diagnosis, as glomerulonephritis, hemolytic uremic syndrome, and acute viral hepatitis can present with similar laboratory and clinical findings, including elevated liver enzyme levels, jaundice, and renal dysfunction.

Co-infections with viruses such as EBV, CMV, and HIV can further obscure the diagnosis owing to overlapping immunological and systemic manifestations [29,30].

Table 2 summarizes the main differential diagnoses.

### 4.8. Complications

VL in children can lead to severe complications that often overlap with other diseases, making an accurate diagnosis challenging. Severe complications include post-kala-azar dermal leishmaniasis (PKDL), secondary HLH, disseminated intravascular coagulation (DIC), and hepatic failure [7].

PKDL is a complication that may develop following VL even when successfully treated. It is characterized by skin lesions, typically appearing months to years after VL treatment. These lesions range from macules to nodules and plaques, often located on the face, arms, and trunk, and are a major diagnostic challenge owing to their resemblance to other dermatologic conditions. The pathogenesis of PKDL is still not fully understood, but it is believed to be related to a combination of immunological factors and residual parasites in the skin [30].

HLH is a rare but potentially fatal complication caused by excessive cytokine production and abnormal proliferation of cytotoxic lymphocytes and histiocytes, leading to hemophagocytosis. It is a heterogeneous disorder in which defects in natural killer cells and T-cell granule-mediated cytotoxic function result in an uncontrolled inflammatory immune response and cytokine storm, ultimately causing tissue damage and multi-organ failure. VL is recognized as a trigger for secondary HLH along with infections such as EBV, CMV, and HIV. In children with VL, HLH can worsen prognosis, especially when associated with immunosuppressive treatments or viral co-infections. It typically presents as fever, hepatosplenomegaly, cytopenia, and elevated ferritin levels. HLH can manifest during the acute phase of VL or after treatment initiation, particularly when the therapeutic response is delayed or inadequate [31,32].

DIC is a severe complication that can occur in patients with advanced disease, and as the disease progresses, hepatic failure may develop, causing spontaneous bleeding. It also involves the widespread activation of the coagulation system, leading to both excessive clotting and bleeding. DIC in visceral leishmaniasis is often associated with severe systemic inflammation, multiorgan failure, and poor prognosis.

Renal impairment due to VL includes interstitial and glomerular dysfunction due to immune complex deposition, which contributes to kidney damage. These complications further complicate the clinical picture and increase the risk of misdiagnosis.

Co-infections with viruses, such as Parvovirus B19 and Influenza, can further complicate VL. Parvovirus B19 can induce bone marrow suppression, leading to severe anemia, while the Influenza virus can exacerbate respiratory symptoms and increase susceptibility to secondary bacterial infections. These overlapping clinical features necessitate a comprehensive diagnostic approach to differentiate between VL complications and other potential diagnoses [33].

Our case series highlights the clinical complexity of VL in pediatric patients, particularly regarding its complications. One patient (Case 4) developed early signs of secondary HLH, evidenced by persistent fever, cytopenia, hepatosplenomegaly, hyperferritinemia, and hypertriglyceridemia, requiring close monitoring and targeted therapy. Another case was complicated by Parvovirus B19 coinfection, which contributed to severe anemia and prolonged the diagnostic process. These viral co-infections can delay diagnosis, worsen prognosis, and complicate management.

## 5. Limitations

Our case series provides valuable insights into the clinical presentation and diagnostic challenges of VL in pediatric patients. However, this study had several limitations. First, the small sample size (five cases) limits the generalizability of our findings. While our cases illustrate the heterogeneity of VL presentation, a larger cohort is needed to better define the epidemiological and clinical spectrum of the disease in children.

Second, despite a comprehensive diagnostic workup, variability in laboratory investigations performed among the cases may have influenced the completeness of our analysis. For example, in Case 3, limited serological testing was conducted, which may have led to an underestimation of potential co-infections. Furthermore, the lack of standardized diagnostic protocols increases the risk of underestimating potential differential diagnoses.

Another limitation is the retrospective nature of the analysis, which may have introduced selection bias. Our patients were referred to a tertiary center because of diagnostic uncertainty, potentially excluding milder or self-limiting VL cases. This referral bias might have overrepresented the severe forms of VL, including cases complicated by HLH (as in Cases 3 and 4).

Environmental and epidemiological factors also present challenges in fully assessing the transmission risk. While some patients had a clear exposure history (e.g., case 4, where the child’s family dogs had reported canine leishmaniasis), others resided in urban areas with no known vector exposure. This variability highlights the difficulty in defining precise risk factors, particularly in low-endemic settings.

## 6. Conclusions

Despite advancements in treatment and diagnostics, VL remains a neglected disease, even in high-income nations, with an increasing incidence. Major challenges include high treatment costs and rising drug resistance to antimonials in endemic regions, which pose risks of importation. Moreover, dogs are the primary reservoirs of *L. infantum*, and controlling this reservoir remains a major challenge, especially in regions with large populations of stray or unvaccinated dogs. Integrated public health and veterinary interventions are essential to limit zoonotic transmission. Furthermore, ongoing climate change may alter sandfly habitats, potentially increasing the number of cases in nonendemic areas. Thus, VL is no longer confined to developing countries. Its emergence in countries such as Italy underscores the need for global awareness and integrated public health strategies. Owing to their unique clinical and therapeutic challenges, pediatric cases require special attention. Bridging the gap between high- and low-income regions in terms of treatment accessibility and vector control is essential for achieving the WHO’s goals of eliminating VL as a public health problem. Future strategies should focus on developing affordable oral treatments and effective vaccines. Surveillance systems in Western countries must integrate refugee health programs to detect and treat imported cases early.

## Figures and Tables

**Table 1:** The table presents the laboratory results from blood tests performed on the five patients diagnosed with visceral leishmaniasis.

	Normal Range	Case 1	Case 2	Case 3	Case 4	Case 5
Value upon admission (worse value) [discharge value]						
GOT, U/L	0–40	45 (45) [29]	59 (59) [46]	37 (46) [36]	521 (521) [180]	51 (154) [40]
GPT, U/L	0–40	12 (12) [9]	13 (38) [36]	26 (26) [20]	259 (259) [138]	31 (36) [27]
LDH, U/L	192–321	472 (472) [447]	295 (373) [283]	358 (358) [350]	630 (630) [339]	295 (295)
Triglycerides, md/dL	64–122	121 (121) [102]	359 (574) [574]	317 (555) [347]	252 (1287) [310]	/
Ferritin, ng/mL	20–200	266 (268) [268]	643 (1117) [297]	201 (221) [178]	2409 (2409) [196]	393 (569) [479]
Albumin, mg/dL	3800–5400	3570 (2808) [3882]	2328 (2328) [4085]	3107 (2737) [3680]	3177 (2945) [3242]	2497 (2497) [3869]
Hemoglobin, g/dL	12–14	7.7 (6.1) [9.7]	6.1 (6.1) [10.4]	7.9 (7.0) [9.6]	9.5 (8.4) [8.5]	5.6 (5.6) [9.4]
RBC, cell × 10^6^/μL	4.1–5.5	3.05 (2.50) [3.74]	2.88 (2.74) [4.19]	3.60 (3.10) [3.94]	4.08 (3.63) [3.88]	2.67 (2.67) [3.79]
WBC, cell × 10^3^/μL	4.6–13.7	2.34 (2.34) [3.77]	1.24 (1.06) [4.58]	2.96 (2.35) [6.74]	8.13 (6.32) [7.20]	1.16 (1.16) [3.29]
Platelets cell × 10^3^/μL	150–450	60 (56) [90]	75 (50) [330]	51 (51) [171]	100 (82) [195]	75 (62) [116]
CRP, mg/dL	0–0.46	3.69 (6.60) [0.71]	6.67 (6.76) [0.83]	5.83 (5.83) [0.78]	1.17 (1.58) [<0.46]	16.08 (20.94) [2.09]
Immunoglobulin A, mg/dL	20–100	80	23	23	109	98
Immunoglobulin G, mg/dL	450–1350	1380	1367	1440	1881	2937
Immunoglobulin M, mg/dL	20–145	113	148	29	169	280

**Table 2:** The table summarizes the key clinical and laboratory features that differentiate visceral leishmaniasis from other diseases with similar presentations. Each condition listed is accompanied by distinguishing factors, including symptomatology, laboratory findings, and relevant diagnostic tests.

Condition	Clinical Features	Diagnostic Tests	Key Differentiating Points from VL
**Lymphoma**	Persistent fever, lymphadenopathy, hepatosplenomegaly, weight loss, night sweats	Lymph node biopsy, PET/CT, immunophenotyping	Absence of parasites in bone marrow aspirate; firm, fixed lymph nodes
**Leukemia**	Fever, pallor, petechiae, hepatosplenomegaly, bone pain, severe pancytopenia	Peripheral blood smear, immunophenotyping, bone marrow biopsy, genetic tests	Circulating blasts in blood and bone marrow; no *Leishmania* seen in histology
**Hemophagocytic lymphohistiocytosis**	Persistent fever, cytopenia, hyperferritinemia, hepatosplenomegaly, coagulopathy, hypertriglyceridemia	Elevated ferritin, soluble CD25, high triglycerides, genetic testing for primary HLH	HLH can be secondary to VL, but primary HLH is associated with genetic mutations (PRF1, UNC13D, STXBP2, etc.)
**Typhoid Fever**	Prolonged fever, abdominal pain, hepatosplenomegaly, relative bradycardia	Blood culture positive for Salmonella typhi, Widal test (low reliability)	Positive blood culture and response to antibiotic therapy; absence of *Leishmania* in bone marrow aspirate
**Severe Dengue**	High fever, petechiae, thrombocytopenia, hepatosplenomegaly, shock in severe cases	Dengue IgM/IgG serology, RT-PCR for Dengue virus	Travel history to endemic area; marked leukopenia and thrombocytopenia; no parasites in bone marrow examination
**Tuberculosis**	Chronic fever, weight loss, night sweats, lymphadenopathy, hepatosplenomegaly	Quantiferon test, sputum culture, chest X-ray	Positive Mycobacterium tuberculosis tests, response to anti-TB therapy
**Brucellosis**	Undulating fever, arthralgia, hepatosplenomegaly, profuse sweating	Blood culture, Wright test, Rose Bengal test	Typical undulating fever, exposure to contaminated animal products
**Malaria**	Intermittent fever, Hepatosplenomegaly, anemia	Peripheral blood smear, rapid antigen test, Plasmodium RT-PCR	Travel to an endemic area, parasites identified in peripheral blood
**Infectious Mononucleosis (EBV, CMV)**	Fever, lymphadenopathy, hepatosplenomegaly, pharyngitis	EBV/CMV IgM/IgG serology, atypical lymphocytosis	Pharyngitis, positive EBV/CMV tests
**Parvovirus B19**	Fever, “slapped cheek” rash, lacy body rash, joint pain, anemia, fatigue	Serology (IgM/IgG), RT-PCR	Characteristic rash, mild fever, transient anemia
**Leptospirosis**	Fever, myalgia, jaundice, renal failure, vasculitis	Leptospira serology, RT-PCR, blood or urine culture	History of exposure to contaminated water, jaundice, vasculitic signs
**Bacterial Endocarditis**	Prolonged fever, petechiae new heart murmurs, septic emboli	Blood cultures, transesophageal echocardiogram	Heart murmurs, septic emboli, positive blood cultures

## Data Availability

The datasets used and analyzed in this study are available from the corresponding author upon reasonable request.

## Abbreviations

The following abbreviations are used in this manuscript:

WHO	World Health Organization
VL	Visceral Leishmaniasis
HIV	Human Immunodeficiency Virus
PED	Pediatric Emergency Department
GE	province of Genoa
SV	province of Savona
IM	province of Imperia
EBV	Epstein–Barr Virus
CMV	Cytomegalovirus
RT-PCR	Real-Time Polymerase Chain Reaction
ISS	National Institute of Health (Istituto Superiore della Sanità)
LAmB	Liposomal Amphotericin B
HLH	Hemophagocytic lymphohistiocytosis
CL	Cutaneous Leishmania
CRP	C-Reactive Protein
PKDL	Post-kala-azar dermal leishmaniasis
DIC	Disseminated intravascular coagulation
